# Factors Associated with Bed-Blocking at a University Hospital (Cantabria, Spain) between 2007 and 2015: A Retrospective Observational Study

**DOI:** 10.3390/ijerph16183304

**Published:** 2019-09-09

**Authors:** Amada Pellico-López, David Cantarero, Ana Fernández-Feito, Paula Parás-Bravo, Joaquín Cayón de las Cuevas, María Paz-Zulueta

**Affiliations:** 1 Care Continuity Coordinator, Área VI SESPA, Urbanización Castañeda s/n. C.P., 33540 Arriondas, Principado de Asturias, Spain; 2 Research Group on Public Economics and Health, GIECONPSALUD, University of Cantabria, Avda de los Castros s/n. C.P., 39005 Santander, Cantabria, Spain; 3 Faculty of Economics, University of Cantabria, Avda. de los Castros s/n. C.P., 39005 Santander, Cantabria, Spain; 4 Nursing Area, Department of Medicine, Faculty of Medicine and Health Sciences, University of Oviedo / ISPA. Avda Julián Clavería s/n. C.P., 33006 Oviedo, Principado de Asturias, Spain; 5 Faculty of Nursing, University of Cantabria, Avda Valdecilla s/n. C.P., 39008 Santander, Cantabria, Spain; 6 IDIVAL, Grupo de Investigación en Enfermería. C/ Cardenal Herrera Oria s/n. C.P., 39011 Santander, Cantabria, Spain; 7 Facultad de Derecho, University of Cantabria, Avda. de los Castros s/n. C.P., 39005 Santander, Cantabria, Spain; 8 IDIVAL, GI Derecho Sanitario y Bioética, GRIDES, C/ Cardenal Herrera Oria s/n. C.P., 39011 Santander, Cantabria, Spain

**Keywords:** length of stay, patient discharge, delayed discharge, bed-blocking, social determinants to health

## Abstract

Current studies on bed-blocking or delayed discharge for non-medical reasons report important variations depending on the country or setting under study. Research on this subject is clearly important as the current system reveals major inefficiencies. Although there is some agreement on the patient-related factors that contribute to the phenomenon, such as older age or a lack of functional ability, there is greater variability regarding environmental or organizational factors. This study sought to quantify the number of cases and days inappropriately spent in hospital and identify patient characteristics and healthcare service use associated with the total length of stay. All cases of delayed discharge were studied at the hospitalization units of a general university hospital in Northern Spain between 2007 and 2015. According to regression estimates, the following characteristics were related to a longer stay: higher complexity through (Diagnosis-Related Group) DRG weight, a diagnosis that implied a lack of functional ability, surgical treatment, having to wait for a destination upon final discharge or return home. After an initial increase, a reduction in delayed discharge was observed, which was maintained for the duration of the study period. Multi-component interventions related with discharge planning can favor a reduced inefficiency with fewer unnecessary stays.

## 1. Introduction

‘Bed-blocking’ is a term which first appeared in the United Kingdom during the late 1950s. Other synonymous expressions are the following: long stays, delayed discharge [1], delayed transfer [2], inappropriate hospitalized days [3], alternate level of care [4] or barrier days [5], used in different contexts. All these terms refer to the fact that “*average bed use is interrupted by patients who stay in hospital for longer than expected*” [1]. A recent review on delayed discharge defined bed-blocking as: “*a period of continued stay after a patient is deemed medically fit to leave hospital but is unable to do so for non-medical reasons*” [6].

Most studies on this subject have been published after 1999, mainly in the European Union and North America. Although this issue mainly concerns tax-funded health care services, delayed discharge also occurs in institutions that are primarily funded by private insurance companies [7]. The United Kingdom is the country which has most extensively studied and published research papers regarding bed-blocking. In the 1970s, some studies already described the relationship of this issue with ageing and noted concerns for the efficiency of attending to a patient at the most appropriate level of care [8]. Currently, delayed discharge is regularly monitored, and is considered a key quality indicator by the UK’s National Health Service. Recent reports in Scotland state a prevalence of 7.8% in the total days of admission during the 2017/2018 period, with a tendency towards improvement compared to the previous period [9]. Nonetheless, regarding the total number of health system users affected by the problem of delayed discharge and the impact this has on the number of avoidable hospital stays, a recent review revealed an important variation in their prevalence rates, ranging from 1.6% to 91.3%, with a weighted mean of 22.8%. This variation is important to note when quantifying expenses [7].

In Spain, a study conducted in 2009 of patients hospitalized in internal medicine wards, found a 3.5% incidence of delayed discharge [10]. Delayed discharge implies an inefficient use of acute hospitalization which, in our setting, leads to an avoidable cost for regional governments responsible for the management of social and health care. Spain has a national healthcare system that provides universal care for all residents, with each region being responsible for organizing and administrating their own healthcare services, which are publicly financed by taxes. Second-level care is long-term care comprising rehabilitation and convalescence or palliative care, which is generally provided by private organizations, in agreement with the public healthcare service. Since 2006, the care system for dependent individuals has been partially financed by both taxes and copayments (whereby users contribute according to their income levels). These services are provided by institutions or home care services, which usually involve informal family caregivers. In Spain, the implementation of a care system for dependent individuals has taken place from 2007 to the present day [11]. Delayed discharge is, therefore, heavily impacted by the level of coordination between the health system and long-term and social care. Thus, the expansion of care for dependency has been shown to help reduce inefficiencies in hospital care, such as the number and frequency of hospitalizations [12]. Cantabria, a region of Northern Spain, is experiencing greater demographic ageing. In 2018, 21.5% of the population was aged 65 years or older. This process of demographic ageing is both a health challenge, as the system must incorporate caring for people with chronic pathologies, as well as a social challenge, to manage long-term dependency [13].

Delayed discharge is associated with characteristics that increase the likelihood of bed-blocking. In the studies consulted, we can differentiate between internal characteristics or characteristics related to the patient, and external characteristics, which are dependent on the health system, the process of care or the individual’s environment. Regarding the overall characteristics of the patient, several studies concede that there is a direct relationship between ageing and delayed discharge [1,3,14,15,16,17,18,19,20,21], including more complex processes [14,17,22], such as, in the case of pathologies associated with a greater probability of functional disability [3,14,17,19,23], cognitive decline [17,18,19,23] and psychiatric pathology [21]. The role of gender is unclear. Certain studies fail to find differences between men and women [22]. However, in other studies, men have a lesser risk of delayed discharge [3] or of being institutionalized after discharge; possibly because of the support provided by caregivers [14].

Among the factors related to the process of care or the setting, there seems to be a greater risk of delayed discharge in the case of emergency admissions [18], admittance to hospitals attending more complex cases [22], admittance managed by surgical services [3,19], changes between supervising specialists during the hospital stay [19], rehabilitation needs [19,23] and discharge to a nursing home [15,16,19]. In general, this is characterized by failure of discharge planning and a shortage of available alternative forms of care [1]. Concerning the context of where the person lives, influential factors for delayed discharge include not having a main caregiver or the impossibility of the caregiver to assume care [10,23,24], as well as living alone or without social support [20]. The place of residence can also have repercussions on this phenomenon. Thus, the study by Holmas et al., in 2013 [22], found there was an incidence of longer hospital stays if the patient lived close to the hospital.

There are several critical consequences of delayed discharge, aside from the basic inefficiency due to the inappropriate use of an acute bed that is not required. The overall negative impact can be linked to delayed surgeries, the appearance of complications, greater mortality rates, and, quantitatively, a negative emotional impact on patients and health professionals [6].

The contributions of this study are, firstly, that it brings to light the prevalence of delayed discharge in our environment and is aimed at both health management and clinicians. In Spain, there have been insufficient studies conducted on this phenomenon, and none of those available have the characteristics or breadth of our research. We have included all the services of a university hospital over a nine-year period. Second, we hope to address the lack of evidence in our setting regarding any influential patient and organizational characteristics of the setting. Finally, these findings may be helpful in identifying necessary and realistic measures for improving this situation in Spain.

The aims of this study were to quantify the number of cases of delayed discharge and inappropriate hospital days and to identify the characteristics of patients and healthcare service utilization associated with bed-blocking over a nine-year period at a university hospital located in the North of Spain.

## 2. Materials and Methods

Consent from the hospital authority was obtained prior to the commencement of this study. Identified-patient-level data was made available to the authors at each individual site and was subsequently collated into a single de-identified dataset for analysis and reporting purposes.

### 2.1. Dataset

The setting for this cross-sectional study was the Valdecilla Hospital in Cantabria (Northern Spain). This general university hospital has 907 staffed acute-care beds and a catchment area comprising 327,000 inhabitants. The Valdecilla Hospital is a hospital of reference for two other local hospitals with a catchment area population of 255,000 people. Data were gathered on all patients hospitalized in any ward of the Valdecilla Hospital between 1 January 2007 and 31 December 2015. The samples consisted of patients for whom the hospital had already issued a discharge date but who continued to occupy acute care beds for non-clinical reasons. Patients were identified from the hospital information management system. The hospital admissions service facilitated access to this data based on patient discharge records belonging to the National Health System Register. These records contain information both concerning the patient, as well as data regarding the hospitalization process.

All patients deemed suitable for discharge from the hospital and experiencing a discharge delay of more than 24 h were included. Patients discharged to other hospitals or home hospitalization services were excluded from the assessment.

### 2.2. Outcome Measures

The total length of stay for these patients comprised two time periods: the length of the appropriate stay (from the admission date to the ready-for-discharge date) and the length of the delayed stay (from the ready-for-discharge date to the actual discharge date). We analyzed the effects on total length of stay as the dependent variable.

Independent variables can be divided into two different levels: patient and care characteristics or social characteristics. The selected variables were based on previous studies with similar aims or methodology [3,19,22]. Concerning the patients, we collected the following demographic and health characteristics: gender, age, Diagnosis-Related Group (DRG), DRG weight and Major Diagnostic Categories (MDC). Diagnosis-Related Group (DRG) is a classification system for episodes of hospitalization according to the patient’s age, gender, circumstances of discharge, principal diagnosis for hospitalization, secondary diagnoses and procedures during hospitalization. For this study, we used version GRD-AP 25.0 [25]. The relative DRG weight represents the average cost of caring for discharges in a specific DRG, regarding the average episode (weight = 1). DRGs may be further grouped into Major Diagnostic Categories (MDCs), which group patients with illnesses of the same body region or system. Care or social characteristics included admission type (planned or emergency care), environment (urban, if the patient lived in an urban area within a 15 km radius of the hospital, and rural if they lived in another district, 15 km or more away or were dependent on other local hospitals), type of hospital service (surgical or medical), discharge destination (long-term care center, home, death, or other destinations, such as nursing homes for the elderly or for dependent patients) and year of discharge, from 2007 to 2015.

### 2.3. Statistical Analysis

All data were analyzed using R 3.6.0 for Windows. Descriptive statistics were calculated using frequencies, means, and standard deviations (SD) in order to describe the characteristics of the sample and the periods of stay. A regression analysis was performed to identify differences in dependent variables according to independent variables (patient, and care or social characteristics) in a univariate analysis. A *p*-value of 0.05 was considered statistically significant. Outliers were excluded from the models. A multivariate model using log-linear regression analysis was performed to determine independent variables associated with a longer stay. Those with statistical significance (*p* ≤ 0.2) were entered into the equation, based on the results of previous univariate analyses. Multicollinearity for the regression analysis was verified by reviewing the values of the variance inflation factor.

## 3. Results

### 3.1. Descriptive Statistics

A total of 3015 patients were identified as receiving a delayed discharge during the study period, resulting in a total of 85,989 days of total stay, divided into 63,870 days of appropriate stay and 21,119 days of delayed stay. The mean duration of total stay was 28.52 days (SD 30.10), the mean duration of appropriate stay was 21.18 days (SD 23.18) and the mean duration of delayed stay was 7.34 days (SD 15.88). Up to 28.7% (CI 27.05–30.31) of cases had a delayed stay of only one day (values not shown in the table).

The general characteristics of cases are summarized in Table 1. The average age of the patients in the sample was 77.28 years (SD 11.95). The proportion of women was 52.1%. The DRG weight was 3.76 on average (SD 11.95). The most common DRGs were those related to stroke (6.1%) and other nervous-system disorders (5.6%), pneumonia (4.1%), and hip procedures (3.3%). Furthermore, according to MDC, the most common were those relating to the nervous (20.8%) and musculoskeletal system (15.4%). In total, 93.0% of the sample were planned admissions, with 71.4% of patients admitted and cared for by medical services (non-surgical). In addition, 77.5% of patients resided at an urban location near the hospital. The year 2008 has the greatest proportion of cases of delayed discharge—14.8% compared to 7.4% in 2014. The percentage of patients who were admitted to a long-term care center after discharge was 77.8%.

The Student’s t-test revealed a statistically significant association between gender and age (values not shown in the table), with a mean age of 74.40 years (SD 12.06) in men and 80.12 years (SD 11.19) in women (*p* < 0.001).

### 3.2. Regression Analysis Results

Univariate analyses are presented in Table 2. Concerning patient characteristics, a statistically significant association (*p* < 0.05) was found with the independent variables: age (longer stay in younger patients, *p* < 0.001), DRG weight (longer with high complexity, *p* < 0.001) and major diagnostic category (longer in the case of disorders of the circulatory system). Concerning care characteristics, a statistically significant association (*p* < 0.05) was found with the following independent variables: service in charge (longer in surgical services *p* < 0.001), year of discharge, longer in 2008 and shorter after 2010 (*p* < 0.001) and discharge destination, for those living farther away (*p* < 0.001) or others (*p* = 0.001).

Multivariate analyses are shown in Table 3. There was no multicollinearity problem. The patient factors which were finally determinant for extending the total stay in cases of delayed discharge (*p* < 0.001), were the greatest weight of the GRD, comprised by: circulation pathologies and being admitted for a surgical service. Concerning external factors with regards the year of discharge, and using 2007 as a reference, (i.e., the first year in the period), a clear and significant increase is observed in the subsequent year, 2008, which then reverts after 2010. Those patients who return to their home or to a nursing home for the elderly or dependent people have longer stays.

## 4. Discussion

Our findings reveal that 3015 patients were identified as having received delayed discharge during the study period. This represents a proportion of 0.93% of the total number of hospital discharges during the study period. The literature on this subject points to important variations in the proportion of cases of delayed discharge, compared to the total number of patients receiving hospital discharge. Our study included patients of all ages and comprised all hospital wards. This is a similar method to the one used in the study by Godden et al. in 2009 of the National Health Service [26], which reported an incidence of 1.6%. However, a Spanish study exclusively based on internal medicine hospitalization [10] reported a proportion of 3.5% of the total number of discharges. In the current study, for the cases of delayed discharge, the mean duration of total stay was 28.52 (SD 30.10). Considering the three stay periods (total, appropriate and delayed stay), the disproportion among these is striking, together with the fact that 28.7% of our sample had a delayed stay of only one day. In other studies, we found a wide variation in the length of stay, ranging from 10 days [3] and 21.8 days, which is similar to our results [27]. Much longer stays have also been reported, such as 379.6 days [18]. Furthermore, the number of cases of only a one-day delay is far higher than other studies consulted, in which 8.1% have been reported [17]. The mean appropriate stay of the cases featured in this study is almost three-times the average length of a hospital stay (equaling 7.44 days), as registered by the regional health system [28]. These findings reveal that, to a certain extent, delayed stays are already implied in appropriate stays. In our region, given the shortage of publicly funded rehabilitation or convalescence beds, the Valdecilla Hospital is allowed to transfer patients to a long-term care center that has an agreement with the public system to provide these resources. Thus, the main discharge destination in this study was the long-term care center. In addition, according to other authors, the mean total stay in delayed discharge depends mainly on the discharge destination [18]. In this context, it is likely that clinicians wait for a bed to become available before declaring that a patient is medically fit or ready for discharge, thus prolonging the supposedly appropriate stay and falsifying the delayed stay. Because of this, it is important to record the clinically fit date, as well as the discharge date, in the patient records [29]. To evaluate and compare the efficiency of different hospitals throughout Spain, for example, the average length of stay (ALOS), is an important outcome variable which should be considered using Data Envelopment Analysis (DEA) programming methodologies to optimize input and output variables. This method is used to assess technical efficiency (output) in hospitals, depending on resource consumption (input) [30]. However, as stated, failure to appropriately register the clinically fit date, distorts these data, causing bias and hampering any possibility of comparison.

Regarding the patient characteristics, the average age of the patients in our sample was 77.28 years (SD 11.95), which is similar to other results of studies carried out in Western countries [4,5,10,17,18,31]. In general, there was a consensus among the studies consulted that patients aged over 75 years undergo a longer total stay [15,16], which is more likely to lead to a delayed discharge [32]. However, in other contexts, such as a study from Korea, the authors reported a mean age of 50.2 years [3]. In our study, no relation was found between the most advanced age and longer stays. This association is also unclear in the available literature. However, we did find studies with results suggesting longer delays in older patients [3,32]. In contrast, other studies found that being of younger age was a predictor of greater delayed discharge [27,33]. In both age groups, problems were observed regarding the lengthened stay. In older patients, there is a greater comorbidity, pluripathology or a possibility of complications during the stay. However, younger patients were less likely to be admitted to post-acute facilities, rehabilitation or convalescence centers or had more complex care needs. Furthermore, injuries or illnesses involving an acute lack of functional ability are less common among younger patients and, as a consequence, their family support networks may be unable to assume care.

In our study, we found a greater proportion of women—52.1%. However, we were unable to demonstrate the impact of gender on the duration of stay. We consulted several studies which noted that delayed discharge affects women more [4,14,18] and similarly stated that women experienced longer delays [3,14]. However, other studies found the opposite, with longer stays among male patients [34]. To better understand the differences in gender in the role of the caregiver in our culture, we examined the relationship between age and gender and found significant differences in age, with men being younger.

In Spain, although a new profile is emerging of male caregivers, family caregivers are still present in society and their profile is that of a woman, who may be a partner or daughter, who is a housewife and with a lower educational level (83.2%), [35]. Our results reflect the relationship between delayed discharge in older women and younger men because of the lack of family caregiver or social support, with different causes being associated with each gender. In general, among elderly women, there is a lack of direct family in the context of progressive dependence while, in men, there can be a lack of family and social support when the situation is unexpected.

In our study, DRG weight is significantly associated with a longer stay, as reported in another study [22]. Additional procedures and secondary diagnoses are quantified in the DRG, which adds to the complexity and can prolong the hospital stay [14]. However, not all cases of delayed discharge are more complex, as it is found that 10.0% of cases (95%CI 8.97–11.14) with a relative weight of less than 1—apparently simple cases—had a longer delayed stay. It is probable that these cases were admitted because of burden or lack of a caregiver, thus using acute care as a buffer, while waiting for an intervention on behalf of social care services.

Regarding the pathologies related to the problem of prolonged stays, when a person is disabled as a result of an illness or injury, demands for care can be high and, therefore, these patients cannot be discharged back home. According to research, the central nervous system [10], musculoskeletal (mainly traumatology) [3,19], and circulatory systems [3] were the most frequent diagnostic categories in delayed discharge. In our sample, respiratory illnesses were also involved, with pneumonia being one of the most common DRGs. Our results regarding the mean stay were similar in specific diagnoses like hip fracture [14,24] or stroke [14]. Any deviations in stay, compared to the usual ALOS for each diagnosis, can point towards the detection of a case of delayed discharge, although this difference is not as clear in relation to the specific medical diagnosis. Receiving a surgical intervention with a specific treatment protocol helped determine when the patient was deemed medically fit and those patients did not benefit more from acute hospitalization. For example, among our cases, the ALOS of cases with DRG 818 (hip replacement) was 16.30 days (SD 10.67). During the study period, the mean of the hospital stay for the same DRG varied between 8.70 and 11.04 days, according to the year of discharge. This result is in agreement with the overall country records which reveal an estimated mean of 10.99 days (SD 6.74) [36]. Greater differences are observed in medical conditions where it is harder to determine the precise moment that the patient is medically fit. Thus, the ALOS of cases with DRG 541—simple pneumonia—was 27.30 days (SD 19.17). This was higher than the hospital mean for the same DRG of between 9.72 and 13.50 days in the years prior to the study period.

Considering the characteristics of the process of care provided to patients, the majority of the patients used in our sample were admitted to hospital via the emergency department, as reported in other studies [18]. Although other authors found that emergency admissions had a shorter stay duration than planned admissions [22], our study did not find differences. It is likely that emergency department patients do not constitute a different group, when compared with planned admissions.

Concerning the place of residence prior to admission, in our region, patients who lived in another district, away from the local hospital of reference, were admitted to the university hospital probably because of more complex diagnoses that could prolong the hospital stay. However, we did not find any relationship with greater stays in the cases of delayed discharge. Other authors found longer stays if the patient lived in another district, was located 15 kms or more from the hospital or depended on other local hospitals. An explanation for this is because the shorter length of stay is due to greater availability of long-term care beds in the area near the hospital [14]. The studies that report the opposite findings, i.e., longer stays for patients who reside in the same hospital area, understand that the hospital was used as a temporary buffer due to the lack of social services [22].

According to previous studies [3], patients undergoing surgery experienced a longer length of delayed discharge. This result is most likely due to the complexity of the surgical intervention in itself and the lack of associated functional capacity.

Throughout the study period, using the year 2007 as a reference to mark the beginning of the observation period, we found significant differences over the years. After an increase in 2008, the situation reverts in 2010, and is subsequently maintained. The study by Holmas et al., 2013 [22], based on the period between 2007 and 2009, also found a significant reduction in stays over the years. In our setting, this phenomenon may be explained due to the progressive implementation of a care system for dependent individuals. After assessing the cases with the greatest level of dependency in 2009, the general population was assessed across the different regions of Spain, with Cantabria being considered one of the most advanced regarding this type of care [11]. The effect registered in the year 2010 may be explained by the impact of greater coordination between health services and long-term care. However, another factor worth considering is the major economic crisis that took place during the same period. In this context, a new profile of male caregivers appeared, the partner or son, retired or unemployed, with primary-level education. The greater availability of caregivers, in this case due to unemployment, may have contributed to reducing the total number of delayed discharge cases and time spent in acute hospitalization services [10,23,24]. Furthermore, after the initial years of the economic crisis, a study similar to ours was conducted in a Spanish hospital [37] that showed improvements in indicators of efficiency and overall productivity because of management measures. This trend may also be a factor in our study and would help explain the improvement observed in the hospitalization periods after 2010.

Regarding the discharge destination, the most frequent (77.8%) was long-term care providing rehabilitation, convalescence and palliative care. In up to 6.6% of cases, the patient died during the delayed stay, which is similar to other studies that found the proportion of deaths to be 4.4% [29]. However, this was a lower rate than a report by Meschi et al., which found rates of 16.3% for bed blockers in a long-term unit [31]. Mortality rates are generally due to patients considered medically appropriate for discharge because therapeutic possibilities have been depleted. However, these patients suffer from a terminal illness and die waiting for discharge to long-stay hospitals with palliative care units. According to the literature, those who are discharged to nursing homes for the elderly or dependent have a longer period of stay [4,15,16,19]. Although other studies found shorter stays in cases where the patient returns to their home, compared to when they are discharged to nursing homes [14,15]. In our study, the impact on hospitalization was also significant and similar in cases in which patients returned to the home, probably because of a need for a structural adaptation, or changes in the family dynamics. In view of the results, several measures may be applicable in our setting to reduce the costs attributed to inappropriate use of hospitalization beds in acute patients. Most of our cases were referred to long-term care. By increasing these resources to intermediate care hospitals, this contributes to reducing the hospital stay without increasing hospital readmissions, representing a more efficient alternative to traditional hospitalization, although without any effect on the community setting [38]. Increasing the number of available beds in nursing homes has an effect, albeit discreet, on the reduction of length of stay [39]. Knowing the related factors, the early planning of hospital discharge [40] has demonstrated positive results on the reduction of hospital stays and readmissions, as well as an increased satisfaction on behalf of patients and family members. Lastly, a recent study defends the results of multi-component interventions at discharge with individual planning, geriatric assessment, support and family/patient education and follow-up after discharge [41].

Our study has several limitations. First, as this was a secondary analysis based on data from the National Health System Register, we selected the variables which tend to be stated in the data based on patient discharge records homogenously, systematically and objectively. In order to identify possible predictors of these delays among a series of variables retrieved from discharge abstracts, the following variables were collected: demographic data, clinical conditions, type of care, and social characteristics. These variables could be included in the analyses; however, it is important to note that, in this study, we were unable to identify characteristics of the patient and their environment which may condition their transition from the hospital to long-term care and social care, such as functional dependency, informal caregiving or living alone. Thus, while these variables may generate endogeneity as they explain the delay in discharge, they have not been measured. This is because, although this information is included in the notes that professionals make in clinical registers, there is no homogenous and systematic register of the same (compared to hospital administration data). For this reason, this data could not be included in the analyses. Second, this study was based on a single institution, meaning that the external validity of these findings is limited with regards to other health systems. In addition, prospective studies on this subject enable the identification of medical discharge dates (when the patient is deemed medically fit), which, ideally, are the same date in which patients can be transferred to a long-term care facility if there are no entrance hindrances/waiting periods. Therefore, our prediction model may not explain a sufficient variation.

## 5. Conclusions

Our results reveal that 0.93% of the total patients studied were identified as being discharged with a delay during the study period. The mean length of appropriate stay was almost three times the mean hospital stay (7.44 days) reported by the regional health system. These findings reveal bias because there is a delay in the appropriate stay. This may indicate that the appropriate stay itself is being prolonged while patients wait for a place to become available. We consider the total length of stay as being the dependent variable.

Regarding the patient characteristics, although a greater proportion of the cases were older than 75 years, there was no relationship between older age and longer stays. Different groups were found when comparing age and gender, identifying two distinct groups: older women and younger men. Having a diagnosis implying a lack of functional ability correlated with a longer stay. In our study, DRG weight is significantly associated with a longer stay. However, we found cases with a relative weight of less than 1 (apparently, simple cases), also with delayed discharge. Most likely, these cases were admitted because of caregiver burden, or the lack of a caregiver, whereby acute care is used as a buffer while waiting for social care.

Concerning external factors, such as the year of discharge, we found longer stays at the beginning of the period, with a tendency for this to improve. This result may be related to the implementation of a system of care of dependent people or changes in family dynamics and hospital management measures related to the economic crisis affecting the country at the time. Finally, concerning the discharge destination, the most frequent destination was long-term care centers providing rehabilitation, convalescence and palliative care. However, it appears that when patients are discharged to their home or to a nursing home, the stay is longer.

Future research should consider variables, such as patient’s level of skill in activities of daily living before, and after, the hospital stay, or circumstances such as the lack of a caregiver, alcoholism or mental disorders in order to provide a more suitable explanation regarding vulnerability issues in these cases. Furthermore, it would be advisable to compare bed-blocking cases with controls in cases where no delays occur, despite sharing similar demographic or clinical characteristics.

## Figures and Tables

**Table 1:** General characteristics of the population under study: periods of stay, gender, age, DRG, MDC, admission, environment, service, year of discharge and destination. Cantabria (Northern Spain), 2007–2015.

				Total Stay (Days)	
		*n* = 3015	%	Mean	SD
**Gender**	Female	1571	52.1	30.40	35.15
	Male	1444	47.9	26.79	24.45
**Age (years) in groups**	≤45	40	1.3	54.45	68.87
	46–60	266	8.8	38.98	40.39
	61–75	720	23.9	32.90	42.14
	76–90	1691	56.1	25.62	19.83
	≥91	298	9.9	21.58	15.72
**DRG ^a^**	14 ^b^	183	6.1	24.59	15.79
	533 ^c^	168	5.6	31.32	19.46
	541 ^d^	125	4.1	27.30	19.17
	211 ^e^	99	3.3	15.06	9.81
	818 ^f^	90	3.0	16.30	10.67
**DRG Weight in groups**	0–1	302	10.0	22.93	27.01
	1.1–2	949	31.5	21.49	19.94
	2.1–4	1105	36.7	25.58	22.36
	≥4.1	659	21.9	46.10	44.67
**MDC ^g^**	Nervous	628	20.8	26.53	22.50
	Musculoskeletal	465	15.4	23.11	26.58
	Respiratory	393	13.0	28.22	35.71
	Circulatory	300	10.0	32.03	24.25
	Digestive	231	7.7	23.93	21.48
**Admission**	Planned	211	7.0	30.01	26.09
	Emergency	2804	93.0	28.41	30.31
**Environment**	Rural ^h^	678	22.5	27.80	28.37
	Urban ^i^	2337	77.5	30.99	35.33
**Service**	Medical	2154	71.4	27.08	28.88
	Surgical	861	28.6	32.12	32.70
**Year of discharge**	2007	372	12.4	33.36	42.46
	2008	443	14.8	38.22	33.64
	2009	374	12.4	27.86	27.01
	2010	362	12.1	23.10	28.80
	2011	396	13.1	24.75	23.91
	2012	296	9.7	24.85	22.11
	2013	275	9.2	27.09	26.17
	2014	227	7.4	23.86	18.17
	2015	264	8.8	28.87	40.47
**Discharge destination**	Long-term care	2377	77.8	26.53	26.63
	Home	412	13.6	38.31	32.97
	Death	198	6.6	28.45	44.00
	Others ^j^	28	0.9	60.79	71.76

^a^ DRG, Diagnosis-Related Group; ^b^ DRG 14, stroke; ^c^ DRG 533, other nervous-system disorders; ^d^ DRG 541, simple pneumonia; ^e^ DRG 211, hip and femur procedures; ^f^ DRG 818, hip replacement; ^g^ MDC, Major diagnostic category; ^h^ Rural: the patient lived in another district over 15 km away from the hospital or was dependent on other local hospitals; ^i^ Urban: the patient lived in an urban area within a 15 km radius of the hospital; ^j^ Nursing home for elderly or dependent people.

**Table 2:** Univariate analyses. Cantabria (Northern Spain), 2007–2015.

	Total Stay (Days)			
		Estimate	CI ^a^	*p*-Value
**Gender**	Female	Reference		
	Male	0.04	(−0.01, 0.10)	0.1
**Age (years)**		−0.01	(−0.01, 0.00)	<0.001
**DRG Weight**		0.04	(0.03, 0.04)	<0.001
**MDC ^b^**	Circulatory	Reference		
	Digestive	−0.33	(−0.46, −0.19)	<0.001
	Musculoskeletal	−0.36	(−0.47, −0.24)	<0.001
	Nervous	−0.17	(−0.27, −0.06)	0.002
	Respiratory	−0.18	(−0.30, −0.07)	0.002
**Admission**	Planned	Reference		
	Emergency	−0.06	(−0.17, 0.05)	0.3
**Environment**	Rural ^c^	Reference		
	Urban ^d^	−0.07	(−0.14, −0.01)	0.3
**Service**	Medical	Reference		
	Surgical	0.14	(0.08, 0.20)	<0.001
**Year of discharge**	2007	Reference		
	2008	0.21	(0.11, 0.32)	<0.001
	2009	−0.10	(−0.21, 0.01)	0.072
	2010	−0.24	(−0.36, −0.13)	<0.001
	2011	−0.22	(−0.33, −0.11)	<0.001
	2012	−0.22	(−0.34, −0.10)	<0.001
	2013	−0.17	(−0.29, −0.05)	0.005
	2014	−0.21	(−0.34, −0.08)	0.001
	2015	−0.15	(−0.27, −0.03)	0.015
**Discharge destination**	Long-term care	Reference		
	Home	0.40	(0.32, 0.48)	<0.001
	Death	−0.03	(−0.14, 0.08)	0.609
	Others ^e^	0.49	(0.20, 0.79)	0.001

^a^ Confidence interval, ^b^ MDC, Major diagnostic category; ^c^ Rural: the patient lived in another district over 15 km away from the hospital or was dependent on other local hospitals; ^d^ Urban: the patient lived in an urban area within a 15 km radius of the hospital; ^e^ Nursing home for elderly or dependent people.

**Table 3:** Multivariate analyses. Cantabria (Northern Spain), 2007–2015.

	Total Stay (Days)			
		Estimate	CI ^a^	*p*-Value
**DRG Weight**		0.04	(0.03,0.04)	<0.001
**MDC ^b^**	Circulatory	Reference		
	Digestive	−0.27	(−0.39, −0,15)	<0.001
	Musculoskeletal	−0.40	(−0.51, −0.29)	<0.001
	Nervous	−0.11	(−0.21, −0.01)	0.027
	Respiratory	−0.08	(−0.19, 0,03)	0.143
**Service**	Medical	Reference		
	Surgical	0.15	(0.08, 0.22)	<0.001
**Year of discharge**	2007	Reference		
	2008	0.17	(0.07, 0.27)	<0.001
	2009	−0.10	(−0.20, 0.01)	0.062
	2010	−0.23	(−0.33, −0,13)	<0.001
	2011	−0.22	(−0.32, −0.11)	<0.001
	2012	−0.20	(−0.31, −0.09)	<0.001
	2013	−0.18	(−0.29, −0.07)	0.002
	2014	−0.17	(−0.29, −0.05)	0.005
	2015	−0.14	(−0.25, −0.02)	0.019
**Discharge destination**	Long-term care	Reference		
	Home	0.33	(0.25, 0.40)	<0.001
	Death	−0.04	(−0.15, 0.06)	0.426
	Others ^c^	0.39	(0.12, 0.67)	0.005

^a^ Confidence interval; ^b^ MDC, Major diagnostic category; ^c^ Nursing home for elderly or dependent people.
